# Curcumin Inhibits Glutamate Release from Rat Prefrontal Nerve Endings by Affecting Vesicle Mobilization

**DOI:** 10.3390/ijms13079097

**Published:** 2012-07-20

**Authors:** Tzu Yu Lin, Cheng Wei Lu, Shu Kuei Huang, Su Jane Wang

**Affiliations:** 1 Department of Anesthesiology, Far-Eastern Memorial Hospital, Pan-Chiao District, New Taipei City 22060, Taiwan; E-Mails: drlin1971@gmail.com (T.Y.L.); drluchengwei@gmail.com (C.W.L.); nskh9450n@yahoo.com.tw (S.K.H.); 2 Department of Mechanical Engineering, Yuan Ze University, Taoyuan 320, Taiwan; 3 Graduate Institute of Basic Medicine, Jen Catholic University, No.510, Zhongzheng Rd., Xinzhuang District, New Taipei City 24205, Taiwan; 4 School of Medicine, Fu Jen Catholic University, No.510, Zhongzheng Rd., Xinzhuang District, New Taipei City 24205, Taiwan

**Keywords:** curcumin, glutamate exocytotic machinery, ERK, synapsin I, prefrontocortical nerve terminals

## Abstract

Curcumin, one of the major constituents of *Curcuma longa*, has been shown to inhibit depolarization-evoked glutamate release from rat prefrontocortical nerve terminals by reducing voltage-dependent Ca^2+^ entry. This study showed that curcumin inhibited ionomycin-induced glutamate release and KCl-evoked FM1-43 release, suggesting that some steps after Ca^2+^ entry are regulated by curcumin. Furthermore, disrupting the cytoskeleton organization using cytochalasin D abolished the inhibitory action of curcumin on ionomycin-induced glutamate release. Mitogen-activated protein kinase kinase (MEK) inhibition also prevented the inhibitory effect of curcumin on ionomycin-induced glutamate release. Western blot analyses showed that curcumin decreased the ionomycin-induced phosphorylation of extracellular signal-regulated kinase 1 and 2 (ERK1/2) and synaptic vesicle-associated protein synapsin I, the main presynaptic target of ERK. These results show that curcumin-mediated inhibition of glutamate release involves modulating downstream events by controlling synaptic vesicle recruitment and exocytosis, possibly through a decrease of MAPK/ERK activation and synapsin I phosphorylation, thereby decreasing synaptic vesicle availability for exocytosis.

## 1. Introduction

**Figure 1:**
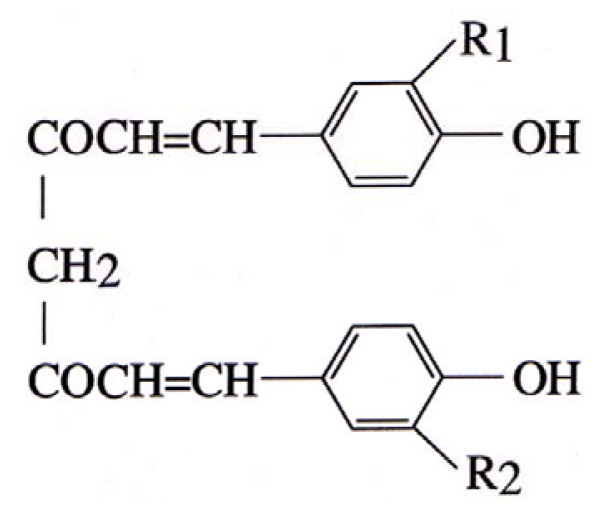
The chemical structure of curcumin.

*Curcuma longa* Linn. is widely used as a yellow coloring agent and spice in numerous foods, such as curry powder and herbal medicine for the treatment of inflammatory conditions, cancer, and acquired immunodeficiency syndrome [1,2]. The therapeutic effects of *Curcuma longa* L. have been postulated to be caused by its active compound curcumin (1,7-bis(4-hydroxy-3-methoxyphenyl)-1,6-heptadiene-3,5-dione, Figure 1), which has many pharmacological properties such as those that inhibit lipid peroxidation or are antioxidant, anti-inflammatory, or anticancer [3–5]. In addition to these properties, numerous studies have demonstrated the neuroprotective effects of curcumin. For example, curcumin attenuates beta-amyloid-, H_2_O_2_^−^, AlCl_3_^−^, and glutamate-induced neurotoxicity [6–9], protects against ischemia-, seizure-, and neurotoxin-induced brain injury [9–13], and ameliorates AlCl_3_ combined with D-galactose-induced learning and memory impairment [9]. However, the mechanisms involved in these neuroprotective effects have yet to be fully elucidated.

Glutamate is a major excitatory neurotransmitter in the brain, and plays an important role in functions such as synaptic plasticity, learning, and memory [14,15]. Excessive release of glutamate induces an increase in intracellular Ca^2+^ levels. This in turn triggers a cascade of cellular responses, including enhanced oxygen free radical production, disturbed mitochondrial function, and protease activation, ultimately leading to neuronal cell death. This type of neuronal damage induced by over-excitation is likely involved in a number of neuropathological conditions, ranging from acute conditions such as stroke, epileptic seizures, traumatic brain and spinal cord injury, to chronic neurodegenerative disorders such as Alzheimer’s disease, Parkinson’s disease, and amyotrophic lateral sclerosis [16–18]. Consequently, if a drug can attenuate glutamate release from nerve terminals, this may have a neuroprotective effect in pathological conditions related to excessive glutamate release.

We have previously demonstrated that curcumin depresses the depolarization-evoked glutamate release from prefrontocortical nerve terminals by reducting presynaptic Ca^2+^ influx [19]. Aside from regulating glutamate release by controlling presynaptic Ca^2+^ entry, there are multiple potential loci for regulating exocytosis downstream from ionic events. In this study, we assessed whether curcumin could also modulate release processes such as synaptic vesicle recruitment, docking, and exocytosis. We found that, by isolating nerve terminals from the rat prefrontal cortex, the modulation of some exocytotic steps after Ca^2+^ influx is involved in the part of the glutamate release inhibited by curcumin. Moreover, this release inhibition is associated with a decrease of MAPK activity and synapsin I phosphorylation, thereby changing cytoskeleton dynamics and increasing vesicle availability.

## 2. Results

**Figure 2:**
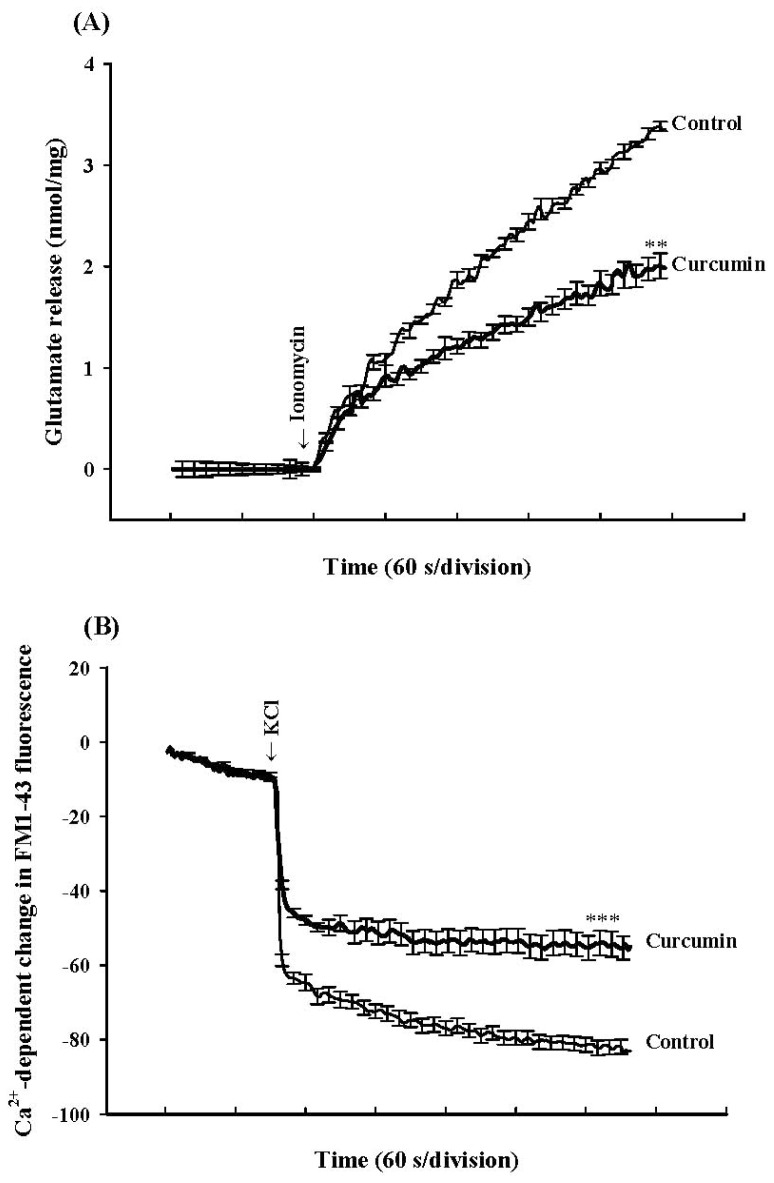
Curcumin inhibites ionomycin-induced glutamate release and KCl-evoked FM 1-43 release in rat prefrontocortical synaptosomes. Synaptosomes were resuspended in incubation medium at a final protein concentration of 0.5 mg/mL and incubated for 3 min before the addition of 1 mM CaCl_2_. Glutamate (**A**) and FM 1-43 (**B**) release were measured under control conditions or in the presence of curcumin (6 μM) added 10 min prior to the addition of ionomycin (5 μM) or KCl (30 mM). Results are means ± SEM of 5–6 independent experiments. *** *p* < 0.001, ** *p* < 0.01, two-tailed Student’s *t* test.

To investigate whether the regulation of glutamate release by curcumin occurred downstream of Ca^2+^ entry, we examined the effect of curcumin on glutamate release induced by ionomycin in rat prefrontal cortex nerve terminals (synaptosomes). The Ca^2+^-selective ionophore ionomycin was used in this study as it has been reported that ionomycin can induce the release of vesicular glutamate independently of voltage-dependent Ca^2+^ channel activation, and therefore ionomycin-induced release reflects the modulation of the release machinery downstream of Ca^2+^ [20]. In Figure 2A, ionomycin (5 μM) caused a control glutamate release of 3.6 ± 0.03 nmol/mg/5 min. In the presence of curcumin (6 μM), ionomycin-induced release of glutamate was inhibited to 2.0 ± 0.1 nmol/mg/5 min (*n* = 6; *p* < 0.01; two-tailed Student’s *t* test), indicating that the curcumin-mediated inhibition of glutamate release is due to direct effects on the exocytotic machinery. In order to further clarify the involvement of the exoctyotic machinery, we examined the effect of curcumin on FM1-43 release. FM1-43 is a lipophilic but membrane-impermeable fluorescent styryl dye. When the styryl dye FM1-43 reversibly partitions into the outer leaflet of exposed plasma membrane, its fluorescence increases. By endocytosis, FM1-43 dye can be taken up into the synaptic vesicles and during subsequent exocytosis, is lost to the extracellular medium, accompanied by a decrease in fluorescence [21]. In Figure 2B, KCl (30 mM) caused a decrease in FM1-43 fluorescence in the presence of CaCl_2_. Notably, this KCl-evoked Ca^2+^-dependent decrease in FM1-43 fluorescence was also significantly inhibited by curcumin (6 μM) (*n* = 5; *p* < 0.001; two-tailed Student’s *t* test).

**Figure 3:**
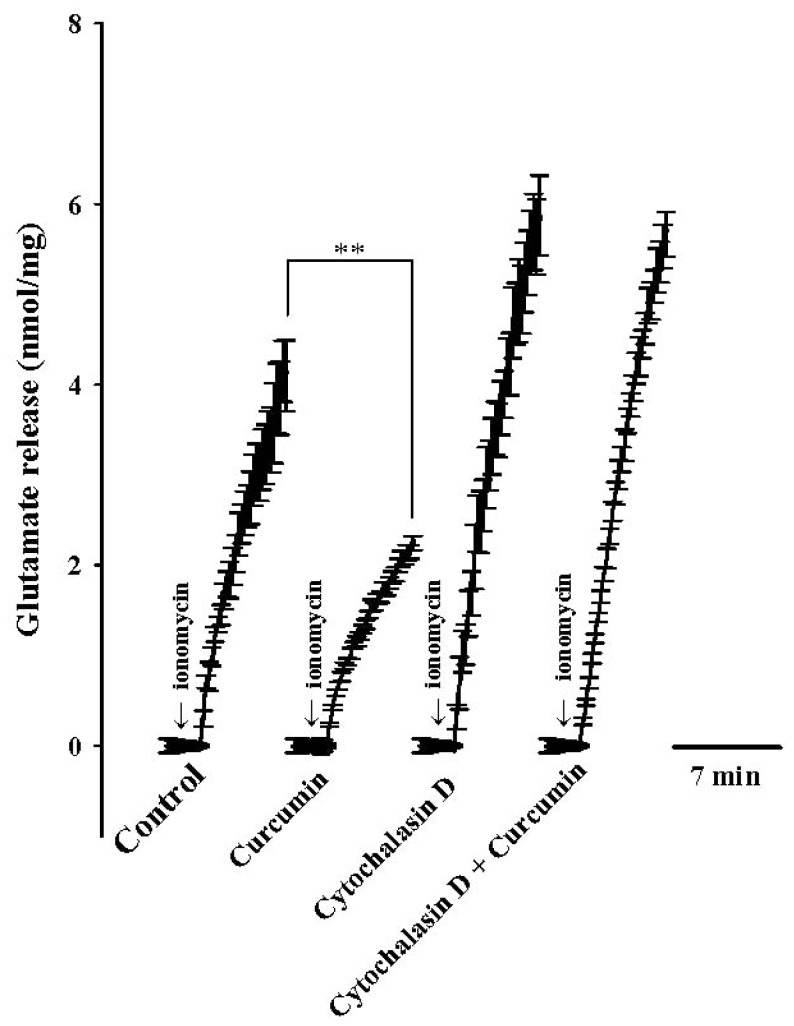
Curcumin-mediated inhibition of ionomycin-induced glutamate release is abolished by cytochalasin D, an inhibitor of actin polymerization. Glutamate release was evoked by ionomycin (5 μM) in the absence (control) or presence of curcumin (6 μM), cytochalasin D (40 μM), cytochalasin D (40 μM) + curcumin (6 μM). Cytochalasin D alone was added 10 min before addition of ionomycin, curcumin was added 10 min after the first addition of cytochalasin D. Results are means ± SEM of 5 independent experiments. ** *p* < 0.01, one-way repeated-measures analysis of variance (ANOVA).

Because actin cytoskeleton disassembly is associated with modulation of the pool of available vesicles and facilitation of glutamate exocytosis [22], it is possible that an alteration in cytoskeleton organization took part in the observed inhibitory action of curcumin on glutamate release. To assess this possibility, we examined the effect of curcumin on ionomycin-induced glutamate release in the presence of membrane-permeant inhibitor of actin polymerization, cytochalasin D. As shown in Figure 3, application of cytochalasin D (40 μM) increased the release of glutamate induced by ionomycin (5 μM) to 5.9 ± 0.4 nmol/mg/5 min (control ionomycin, 4.1 ± 0.4 nmol/mg/5 min; *p* < 0.01; one-way repeated-measures ANOVA). In the presence of cytochalasin D, application of curcumin (6 μM) failed to produce a significant inhibitory effect on ionomycin-induced glutamate release (5.7 ± 0.2 nmol/mg/5 min; *n* = 5; *p* > 0.05; one-way repeated-measures ANOVA). These data provide evidence that a change in vesicle mobilization participates in the curcumin-mediated inhibition of glutamate release.

**Figure 4:**
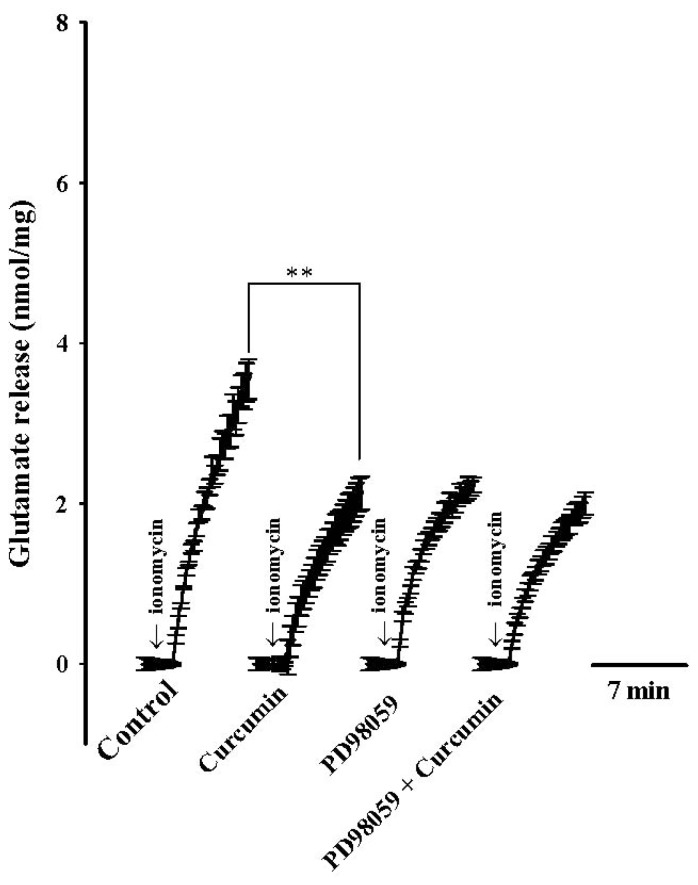
Curcumin-mediated inhibition of ionomycin-induced glutamate release is prevented by mitogen-activated/extracellular signal-regulated kinase kinase (MEK) inhibitor PD98059. Glutamate release was evoked by ionomycin (5 μM) in the absence (control) or presence of curcumin (6 μM), PD98059 (50 μM), PD98059 (50 μM) + curcumin (6 μM). PD98059 alone was added 10 min before addition of ionomycin, curcumin was added 10 min after the first addition of PD98059. Results are means ± SEM of 5 independent experiments. ** *p* < 0.01, one-way repeated-measures ANOVA.

What intracellular signaling was involved in the curcumin-enhanced vesicle availability and glutamate exocytosis? Because mitogen-activated protein kinase (MAPK) has been shown to regulate glutamate release at the presynaptic level [23,24], we investigated whether this cascade participated in the regulation of glutamate release by curcumin. To this end, we used PD98059 to specifically prevent the activation of mitogen-activated/extracellular signal-regulated kinase kinase (MEK) [25], the protein kinase upstream of MAPK. Figure 4 shows that application of PD98059 (50 μM) reduced the ionomycin (5 μM)-evoked glutamate release from 3.6 ± 0.2 nmol/mg/5 min to 2.3 ± 0.1 nmol/mg/5 min, which indicated basal MAPK activity. In the presence of PD98059, the inhibitory effects of curcumin (6 μM)) on ionomycin-induced glutamate release were significantly suppressed (2.0 ± 0.1 nmol/mg/5 min; *n* = 5; Figure 4).

**Figure 5:**
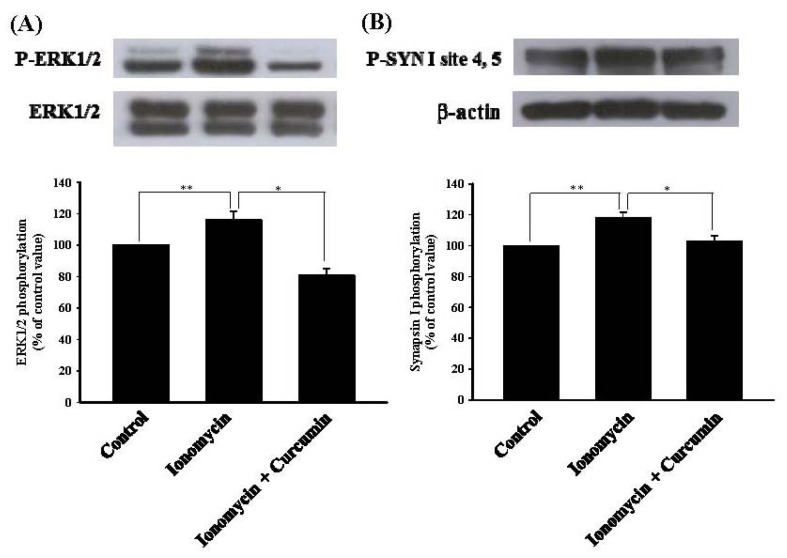
Curcumin decreases ionomycin-induced phosphorylation of extracellular signal-regulated kinase 1 and 2 (ERK1/2) and its presynaptic target synapsin I. Phosphorylation of ERK1/2 (**A**) and synapsin I at mitogen-activated protein kinase (MAPK)-specific sites 4, 5 (P-SYN I site 4, 5) (**B**) was detected in synaptosomal lysates by Western blotting using phosphorylation state-specific antibodies. Purified synaptosomes were incubated for 2 min in HBM that contained 1.2 mM CaCl_2_ at 37 °C in the absence (control) or presence of ionomycin (5 μM), ionomycin (5 μM) + curcumin (6 μM). Curcumin was added 10 min before the addition of ionomycin. Data are expressed as a percentage of the phosphorylation obtained in the controls in the absence of ionomycin stimulation. Results are means ± SEM of 5 independent experiments. ** *p* < 0.01, * *p* < 0.05, one-way repeated-measures ANOVA.

To further confirm that the MAPK signaling cascade was suppressed by curcumin during its inhibition of ionomycin-induced glutamate exocytosis, we performed Western blotting to determine the effect of curcumin on the phosphorylation of extracellular signal-regulated kinases 1 and 2 (ERK) in prefrontal cortical synaptosomes. Figure 5 shows that depolarization of synaptosomes with ionomycin (5 μM) in the presence of 1.2 mM CaCl_2_ increased the phosphorylation of ERK1/2 (116 ± 6%; *p* < 0.01). When synaptosomes were pretreated with curcumin (6 μM) for 10 min before addition of ionomycin, ionomycin-induced phosphorylation of ERK1/2 was markedly decreased to 81 ± 4% (*p* < 0.05; one-way repeated-measures ANOVA; Figure 5A). Furthermore, the effect of curcumin on the phosphorylation of synapsin I, a major presynaptic target of MAPK/EPK, was studied. As shown in Figure 5B, ionomycin (5 μM) increased the phosphorylation of synapsin I in the presence of external Ca^2+^ (118 ± 4%; *p* < 0.01), and this phenomenon was also reduced after curcumin (6 μM) treatment (103 ± 3%; *n* = 5; *p* < 0.05; one-way repeated-measures ANOVA).

## 3. Discussion

Our previous work indicated a correlation of the inhibitory effect of curcumin on glutamate release with a suppression of presynaptic Ca^2+^ entry; however, the possibility remains that curcumin could affect targets downstream from Ca^2+^ entry to depress glutamate release. To investigate this eventuality, in this study we examined the effect of curcumin on the release of glutamate induced by ionomycin. The Ca^2+^-selective ionophore ionomycin causes a direct increase in intrasynaptosomal Ca^2+^ levels and triggers a release of a neurotransmitter without depolarization or Ca^2+^ channel activation [20]. This protocol, therefore, allows the assessment of only modulatory influences directly affecting synaptic vesicle trafficking and exocytosis, without the involvement of the upstream ion-channel function. We found that glutamate release induced by ionomycin was inhibited by curcumin. Furthermore, using an exocytosis assay with the FM1-43, curcumin reduced FM1-43 release evoked by depolarization. Therefore, these results indicate that curcumin has a direct inhibiting role in some exocytotic steps, potentially, at the level of synaptic vesicle trafficking within the nerve terminal.

Regarding the neurotransmitter exocytotic process, actin cytoskeleton plays a vital role in controlling the number of synaptic vesicle available. Disassembling actin cytoskeleton was demonstrated to enhance synaptic vesicle availability and glutamate release [22,26]. Thus, a change in vesicle mobilization may also contribute to the release inhibition of curcumin. In this study, the possibility that curcumin depresses glutamate release through interactions with cytoskeleton components was examined using cytochalasin D, which disrupts filamentous actin, causing disassembly of the cytoskeleton [27,28]. We observed that cytochalasin D increased ionomycin-induced glutamate release, likely because of an increase in the number of synaptic vesicle available for exocytosis. More importantly, in the presence of cytochalasin D, the inhibitory effect of curcumin on ionomycin-induced glutamate release was abolished, suggesting that both cytochalasin D and curcumin act at the same presynaptic site. Therefore, interference in disassembly of the cytoskeleton, which decreases synaptic vesicle availability, seems to be involved in the observed inhibitory effect of curcumin on glutamate exocytosis.

Vesicle mobilization is a complex phenomenon that is regulated by various protein kinases. An essential kinase is MAPK, highly expressed at the presynaptic level [29,30]. Previous studies have demonstrated that MAPK can increase vesicle availability and glutamate exocytosis by phosphorylating synapsin I, a major substrate for MAPK and a presynaptic protein regulating the vesicle cycle and neurotransmitter release [30,31]. Under resting conditions, synapsin I anchors synaptic vesicles to cytoskeletal elements, whereas once phosphorylated by MAPK, it dissociates from synaptic vesicles, thereby increasing more vesicles available at the active zone for neurotransmitter release [30,32]. In this study, we suggest that the suppression of MAPK/ERK-dependent synapsin I phosphorylation and the consequent decreased availability of synaptic vesicles is involved in the observed curcumin-mediated inhibition of glutamate release. This hypothesis is supported by the following results: (1) the MEK (MAP kinase kinase) inhibitors abolished the inhibitory effect of curcumin on the ionomycin-evoked vesicular glutamate release; and (2) curcumin reduced the ionomycin-induced phosphorylation level of ERK1/2 and synapsin I at MAPK-specific sites 4 and 5. However, aside from synapsin I, the possible involvement of other synaptic proteins should be considered. Synapsin II and synapsin III, for example, are reported to be phosphorylated by MAPK [33,34].

## 4. Experimental Section

### 4.1. Materials

FM1-43 was obtained from Invitrogen (Carlsbad, CA, USA). Cytochalasin D and PD98059 were obtained from Tocris Cookson (Bristol, UK). Curcumin, ionomycin, sodium dodecyl sulfate (SDS), and all other reagents were obtained from Sigma-Aldrich Co. (St. Louis, MO, USA).

### 4.2. Animals and Synaptosomal Preparation

Adult male rats (Sprague-Dawley, 150–200 g) (*n* = 31) were housed at constant temperature (22 ± 1 °C) and relative humidity (50%) under a regular light-dark schedule (light 7:00 AM to 7:00 PM). Food and water were freely available. The experimental procedures were approved by the Institutional Animal Care and Use Committee of Fu Jen catholic University. The animals were killed by decapitation, and the prefrontal cortex were rapidly removed at 4 °C. Synaptosomes were prepared as described previously [35]. Briefly, the prefrontal cortex from 2-month-old male Sprague–Dawley rats was isolated and homogenized in a medium that contained 320 mM sucrose, pH 7.4. The homogenate was spun for 2 min at 3000 g (5000 rpm in a JA 25.5 rotor; Beckman Coulter, Inc., Brea, CA, USA) at 4 °C, and the supernatant was spun again at 14,500 g (11,000 rpm in a JA 25.5 rotor) for 12 min. The pellet was gently resuspended in 8 mL of 320 mM sucrose, pH 7.4. Two milliliters of this synaptosomal suspension was added to 3 mL Percoll discontinuous gradients that contained 320 mM sucrose, 1 mM EDTA, 0.25 mM DL-dithiothreitol, and 3, 10 and 23% Percoll, pH 7.4. The gradients were centrifuged at 32,500 g (16,500 rpm in a JA 20.5 rotor) for 7 min at 4 °C. Synaptosomes placed between the 10 and 23% percoll bands were collected and diluted in a final volume of 30 mL of HEPES buffer medium (HBM) that consisted of 140 mM NaCl, 5 mM KCl, 5 mM NaHCO_3_, 1 mM MgCl_2_·6H_2_O, 1.2 mM Na_2_HPO_4_, 10 mM glucose, and 10 mM HEPES (pH 7.4), before centrifugation at 27,000 g (15,000 rpm in a JA 25.5) for 10 min. The pellets thus formed were resuspended in 3 mL of HBM, and the protein content was determined using a Bradford Protein Assay Kit (Bio-Rad, Hercules, CA, USA), based on the method of Bradford (1976) [36], with BSA as a standard. 0.5 mg of synaptosomal suspension was diluted in 10 mL of HBM and spun at 3000 g (5000 rpm in a JA 20.1 rotor) for 10 min. The supernatants were discarded, and the synaptosomal pellets were stored on ice and used within 4–6 h. The homogeneity and morphology of Percoll-purified synaptosomes have been previously characterized [37,38]. Specifically, the composition of this preparation contains synaptosomes and membrane vesicles. The purity of this synaptosomal preparation is approximately 60.3% [37].

### 4.3. Glutamate Release

Glutamate release from purified prefrontal cortical synaptosomes was monitored online, with an assay that employed exogenous glutamate dehydrogenase (GDH) and NADP^+^ to couple the oxidative deamination of the released glutamate to the generation of NADPH detected fluorometrically [39]. Synaptosomal pellets were resuspended in HBM that contained 16 μM BSA and incubated in a stirred and thermostatted cuvette maintained at 37 °C in a Perkin-Elmer LS-50B spectrofluorimeter. NADP^+^ (2 mM), GDH (50 units/mL) and CaCl_2_ (1 mM) were added after 3 min. Other additions before depolarization were made as described in the figure legends. After a further 10 min of incubation, ionomycin (5 μM) was added to stimulate glutamate release. Glutamate release was monitored by measuring the increase of fluorescence (excitation and emission wavelengths of 340 and 460 nm, respectively) caused by NADPH being produced by oxidative deamination of released glutamate by GDH. Data were accumulated at 2-s intervals. A standard of exogenous glutamate (5 nmol) was added at the end of each experiment, and the fluorescence response used to calculate released glutamate was expressed as nanomoles glutamate per milligram synaptosomal protein (nmol/mg).

### 4.4. Styryl Dye Release

Synaptic vesicle fusion with the plasma membrane was measured using release of the fluorescent dye FM1-43, as described previously [21]. In brief, synaptosomes (0.5 mg/mL) were incubated in HBM with 1.2 mM CaCl_2_ for 2 min at 37 °C in a stirred test tube. FM1-43 (100 μM) was added 1 min before stimulation with 30 mM KCl. After 3 min of stimulation to load FM1-43, synaptosomes were washed twice in HBM that contained 1.2 mM CaCl_2_ and 1 mg/mL BSA to remove non-internalized FM1-43. Synaptosomes were then resuspended in 2 mL of HBM (plus 1.2 mM Ca^2+^), and incubated in a stirred and thermostatted cuvette maintained at 37 °C in a Perkin-Elmer LS-50B spectrofluorimeter. Release of accumulated FM1-43 was induced by the addition of 30 mM KCl, and measured as the decrease in fluorescence upon release of the dye into solution (excitation 488 nm, emission 540 nm). Data points were obtained at 2-s intervals, and data presented as the Ca^2+^-dependent decrease in FM1-43 fluorescence. Any drugs were added after the dye-loading procedure, and the synaptosomes were preincubated with curcumin for 10 min before depolarization with KCl.

### 4.5. Western Blot

Synaptosomes (0.5 mg protein/mL) were lysed in ice-cold Tris–HCl buffer solution, pH 7.5, that contained 20 mM Tris–HCl, 1% Triton, 1 mM EDTA, 1 mM EGTA, 150 mM NaCl, 2.5 mM sodium pyrophosphate, 1 mM β-glycerophosphate, 1 mM phenylmethanesulfonyl fluoride, 1 mM sodium orthovanadate and 1 μg/mL leupeptin. The lysates were sonicated for 10 s and then centrifuged at 13,000 g at 4 °C for 10 min. Equal amounts of samples were separated by electrophoresis on 7.5% SDS-PAGE, and then transferred to nitrocellulose membranes. The membranes were blocked with Tris-buffered saline (TBS) that contained 5% low-fat milk and incubated with the following primary polyclonal antibodies: anti-phospho-ERK1/2, 1:2000, anti-ERK1/2, 1:1000, anti-phospho-synapsin-I (Ser^62^/Ser^67^), 1:1000, β-actin, 1:500). After incubation with appropriate peroxidase-conjugated donkey anti-rabbit IgG secondary antibodies (1:3000), protein bands were detected by using the ECL chemiluminescence system (Amersham, Buckinghamshire, UK). An aliquot of samples was loaded and probed with anti-β-actin antibody for detection of β-actin as a loading control. Films were scanned using a scanner and the level of phosphorylation was assessed by band density, which was quantified by densitometry.

### 4.6. Statistical Analysis

Cumulative data were analyzed in Lotus 1-2-3 and MicroCal Origin. Data are expressed as mean ± SEM. To test the significance of the effect of a drug versus control, a two-tailed Student’s *t* test was used. When an additional comparison was required (such as whether a second treatment influenced the action of curcumin), a one-way repeated-measures analysis of variance (ANOVA) was computed. *p* < 0.05 was considered to represent a significant difference.

## 5. Conclusions

This study demonstrated that, in rat prefrontocortical nerve terminals, part of the glutamate release inhibited by curcumin is due to the direct modulation of the release machinery, possible through decreasing MAPK/ERK activation and synapsin I phosphorylation to affect actin cytoskeleton dynamics. This finding may provide more information on the mechanism underlying the inhibitory effect of curcumin on glutamate release. Overall, our present and previous [19] data suggest that curcumin-mediated inhibition of glutamate release may involve modulation of Ca^2+^ entry, as well as downstream events controlling synaptic vesicle trafficking.
